# Consolidation and performance gains in plasma-sintered printed nanoelectrodes†

**DOI:** 10.1039/d3na00293d

**Published:** 2023-07-11

**Authors:** Lukas F. Engel, Lola González-García, Tobias Kraus

**Affiliations:** a INM – Leibniz Institute for New Materials, Campus D2 2 66123 Saarbrücken Germany lola.gonzalez-garcia@leibniz-inm.de +49 (0)681-9300-269; b Department of Materials Science and Engineering, Saarland University, Campus D2 2 66123 Saarbrücken Germany; c Colloid and Interface Chemistry, Saarland University, Campus D2 2 66123 Saarbrücken Germany tobias.kraus@leibniz-inm.de +49 (0)681-9300-389

## Abstract

We report on the unusual, advantageous ageing of flexible transparent electrodes (FTEs) that were self-assembled from oleylamine-capped gold nanospheres (AuNPs) by direct nanoimprinting of inks with different particle concentrations (*c*_Au_ = 3 mg mL^−1^ to 30 mg mL^−1^). The resulting lines were less than 2.5 μm wide and consisted of disordered particle assemblies. Small-Angle X-ray Scattering confirmed that particle packing did not change with ink concentration. Plasma sintering converted the printed structures into lines with a thin, electrically conductive metal shell and a less conductive hybrid core. We studied the opto-electronic performance directly after plasma sintering and after fourteen days of storage at 22 °C and 55% *rH* in the dark. The mean optical transmittance *T̄*_400–800_ in the range from 400 nm to 800 nm increased by up to ≈ 3%, while the sheet resistance *R*_sh_ strongly decreased by up to ≈ 82% at all concentrations. We correlated the changes with morphological changes visible in scanning and transmission electron microscopy and identified two sequential ageing stages: (I) post-plasma relaxation effects in and consolidation of the shell, and (II) particle re-organization, de-mixing, coarsening, and densification of the core with plating of Au from the core onto the shell, followed by solid-state de-wetting (ink concentrations *c*_Au_ < 15 mg mL^−1^) or stability (*c*_Au_ ≥ 15 mg mL^−1^). The plating of Au from the hybrid core improved the FTEs' Figure of Merit FOM = *T̄*_400–800_·*R*_sh_^−1^ by up to ≈ 5.8 times and explains the stable value of ≈ 3.3%·*Ω*_sq_^−1^ reached after 7 days of ageing at *c*_Au_ = 30 mg mL^−1^.

## Abstract 2

Oleylamine-capped Au nanoparticles were imprinted and plasma-sintered, forming flexible transparent electrodes with a closed Au shell and hybrid core. They consolidate with time, increasing the electrode figure of merit up to ≈5.8 times, decreasing sheet resistance.

## Graphical abstract

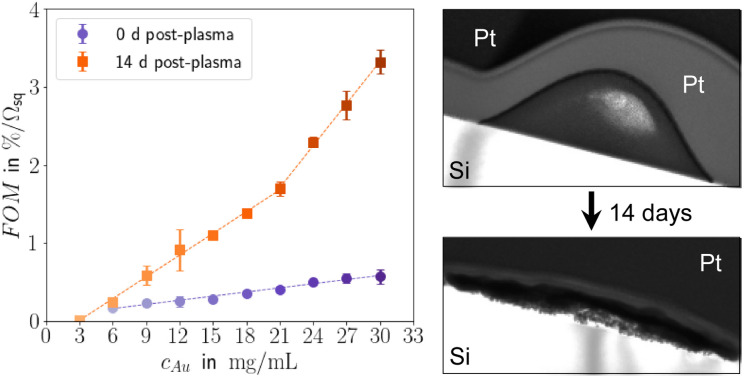

## 1. Introduction

Flexible and transparent electrodes (FTEs) are indispensable components of modern opto-electronics such as foldable smartphones or wearables.^1^ Metal grids with (sub-)micron line widths allow to combine mechanical flexibility and optical transparency with electrical conductance.^2^ Micron-scale conductive metal lines are sufficiently conductive for many applications at areal fractions that let most light pass through. A useful figure of merit of such an electrode is the ratio between optical transmittance and sheet resistance.^3^ It can be tailored to the application by varying the grid pattern, line dimensions, or metal type, for example.^4^

Direct nanoimprinting of colloidal metal inks is an established route to metal grid FTEs.^5^ Printing is feasible in a single, roll-to-roll-compatible step under ambient conditions.^6^ It is possible to print at the sub-micron resolution required for ultra-high definition displays.^7^ Advanced printing processes use a patterned polydimethylsiloxane (PDMS) elastomer stamp that patterns a colloidal metal ink when it meets the substrate. The stamp absorbs the solvent and/or its vapors, the colloids concentrate and self-assemble inside the stamp's cavities, and percolating networks form that follow the stamp's features.^8^

Ko *et al.*^9^ and Park *et al.*^10^ used such a process and nanoimprinted Au nanospheres with a core diameter of *d*_c_ ≈ 1 nm to 3 nm and strongly bound hexanethiol ligands, dispersed in α-terpineol. Kister *et al.*^7^ employed Au nanospheres with *d*_c_ ≈ 3.2 nm and weakly bound dodecylamine (C12) ligands, dispersed in cyclohexane. Maurer *et al.*^8^ imprinted ultra-thin Au nanowires with *d*_c_ ≈ 1.6 nm, lengths above 1.6 μm, and a weakly bound oleylamine (OAm, C18) ligand shell, dispersed in cyclohexane. Park *et al.*^11^ worked with Ag nanospheres with a core diameter of *d*_c_ ≈ 3 nm to 7 nm and dodecylamine (C12) ligands, dispersed in a mixture of toluene and α-terpineol. Agrawal and Garnett^12^ used Ag nanocubes with a core edge length of ≈75 nm and a polyvinylpyrrolidone (PVP) ligand shell, dispersed in ethanol, to form monocrystalline structures.^13^

Nanoimprinting is usually followed by a densification step. For example, colloidal metal typically contains electrically insulating ligands that have to be removed after imprinting.^14^ Thermal, photonic, or plasma-based processes are commonly used for ligand removal and sintering.^15^ Nanoscale colloids can be plasma-sintered at temperatures far below the melting point of the bulk metal.^16^ Plasma sintering avoids softening of the polymer substrate, *e.g.* polyethylene terephthalate (PET) with *T*_g_ ≈ 75 °C.^17^ Oxygen-containing plasma removes organic ligands primarily through oxidation, while inert plasma acts by sputtering.^18^ Both accelerate surface diffusion on metals and cause sintering.^19^ Shaw *et al.*^20^ showed that low-pressure O_2_ plasma penetrates 440 nm thick layers of ceramic nanospheres (ZrO_2_, core diameter *d*_c_ ≈ 3.7 nm, trioctylphosphine oxide ligand shell) and removes virtually all organics while retaining the individual nanospheres. Reinhold *et al.*^21^ and Ma *et al.*^22^ provided evidence for the formation of a conductive Ag shell around an insulating core in a low-pressure Ar plasma for films of printed and drop-casted Ag nanospheres (*e.g.*, *d*_c_ ≈ 23 nm with a gum arabicum ligand shell). Engel *et al.*^23^ reported the formation of a conductive gold shell on top of an oleylamine-gold core for nanoimprinted lines of ultra-thin Au nanowires (AuNWs, *d*_c_ ≈ 1.7 nm) or of Au nanospheres (AuNPs, *d*_c_ ≈ 3.7 nm) in a low-pressure H_2_/Ar plasma.

The last three reports find a strongly improved conductance immediately after plasma treatment. Few authors report on the stability of this improvement or on the efficiency of metal use. It is known that thermally annealed random networks of Ag nanowires degrade as a function of temperature, humidity, light, and electric current.^24–29^ Such electrodes may fragment into spheres upon heating due to the Rayleigh–Plateau instability^30^ and are prone to failure due to electromigration. They are susceptible to UV light and chemical corrosion (sulfides, oxygen, and acids), which can be prevented by protective coatings that reduce surface diffusivity and the Rayleigh–Plateau instability. Maurer *et al.*^31,32^ reported that dip-coated layers (up to 10 nm thick) of AuNWs on glass substrate degraded within hours at room temperature, unless they were coarsened by a H_2_/Ar plasma. Annealing resulted in de-wetting, which accelerated with growing temperature and decreasing film thickness. Engel *et al.*^33^ examined the stability of grid-like FTEs (up to ≈ 275 nm thick) imprinted from AuNWs at different Au concentrations *c*_Au_ on PET foil and identified ageing by solid-state de-wetting of the shell, de-mixing of the hybrid core, and collapse of the shell. All reports indicated a degradation with time that has to be prevented *via* additional coatings or other stabilizing measures.

Here, we show that the degradation of plasma-treated metal grid FTEs is connected to the porosity of the conductive shell and the residual organics inside their hybrid core. We printed FTEs using gold nanospheres (AuNPs) at different concentrations to obtain parallel lines. Their structures were analyzed *via* Small-Angle X-ray Scattering (SAXS) and the lines were sintered in a H_2_/Ar plasma. Electron microscopy (SEM/TEM) before and after sintering indicated the formation of increasingly smooth lines with growing gold concentration. The mean optical transmittances (*T̄*_400–800_) increased by up to ≈ 3.17% within 14 days after sintering, while the electrical resistance *R*_sh_ strongly decreased by up to ≈ 82.2%, depending on the particle concentration. Detailed analyses of the morphological changes indicated that compact, stable shells formed during plasma sintering. They covered hybrid cores that de-mixed after sintering such that additional metal was plated onto the shell, increasing conductance.

## 2. Materials and methods

### 2.1. Synthesis of Au nanospheres

#### 2.1.1. Chemicals

Tetrachloroauric(iii) acid trihydrate (HAuCl_4_·3H_2_O) was synthesized according to Schubert *et al.*,^34^ oleylamine (Acros Organics, C18 content of about 80–90%) was purchased from Thermo Fisher Scientific GmbH (Schwerte, Germany) and filtered with a 0.45 μm Rotilabo-PTFE syringe filter from Carl Roth GmbH + Co. KG (Karlsruhe, Germany) prior to each usage to remove any oxidized residues, pentane (Sigma Aldrich, for HPLC, ≥99%) and borane *tert*-butylamine complex (Sigma Aldrich, 97%) were obtained from Merck KGaA (Darmstadt, Germany), cyclohexane (ROTISOLV ≥99.9%, GC Ultra Grade) was bought from Carl Roth GmbH + Co. KG (Karlsruhe, Germany), and absolute ethanol (≥99.8%, analytical reagent grade) was procured from Fisher Scientific GmbH (Schwerte, Germany). All chemicals were used without further purification unless explicitly mentioned.

#### 2.1.2. Synthesis

Nanospheres (AuNPs) having a gold (Au) core and an oleylamine (OAm) ligand shell were synthesised at ambient conditions according to Kister *et al.*^7^ using an adapted protocol from Wu *et al.*^35^ In a typical synthesis, 80 mg of *tert*-butylamine borane were dissolved in a mixture of 4 mL pentane and 4 mL OAm to obtain a solution A. Then, 200 mg of HAuCl_4_·3H_2_O were given into a disposable 100 mL glass snap-on vial from Carl Roth GmbH + Co. KG (Karlsruhe, Germany). 16 mL of pentane and 16 mL of OAm were added and the resulting mixture stirred at 500 rad min^−1^ for 45 min to obtain a solution B. Precisely at the end of the 45 min (the waiting time determines the sphere radius), solution A was added into solution B (the color turned immediately dark brown). Then the mixture was stirred for further 60 min.

#### 2.1.3. Purification, dilution and storage

After synthesis, the AuNPs were precipitated by adding twice the reaction volume of absolute ethanol and gently shaking the snap-on vial. Sedimentation was forced in a Rotanta 460 RS centrifuge from Andreas Hettich GmbH & Co. KG (Tuttlingen, Germany) with a swing-out rotor at 3435 rcf for 5 min. The supernatant was removed and the spheres were re-dispersed in *n*-hexane, in the amount which corresponded to the original reaction volume. The precipitation procedure was repeated once, followed by forced sedimentation, but this time for 60 min at 3435 rcf, before the spheres were re-dispersed in cyclohexane to obtain a stock dispersion of *c*_Au_ ≈ 30 mg mL^−1^ (estimated based on HAuCl_4_·3H_2_O weighed in and 100% yield). By diluting the stock dispersion, the other concentrations *c*_Au_ of the AuNP inks were prepared. After synthesis and dilution, all dispersions were stored for 16 h at room temperature before use.

### 2.2. Small-angle X-ray scattering (SAXS)

The AuNP core size *d*_sp_ and the colloidal state of order in the dispersions were determined *via* SAXS. The respective dispersion was filled into a glass mark-tube from Hilgenberg GmbH (Malfeld, Germany) with an inner diameter of 1.5 mm. The tube was sealed with a fast-curing two-component epoxy from R&G Faserverbundwerkstoffe GmbH (Waldenbuch, Germany) to exclude solvent evaporation. Each sample was measured for a total of 30 min. The solvent's background was accounted for by measuring its scattering curve separately and subtracting it from the sample curves. To determine *d*_sp_, fitting was performed using the software SASfit from the Laboratory for Neutron Scattering at Paul Scherrer Institute (Villigen, Switzerland).

The spheres' structural arrangement within the pre-plasma lines was studied in transmission right after imprinting on PET. Measurements were performed at room temperature on ambient air to prevent any influence from drawing vacuum. Each sample was measured for 120 min in total.

The scattering setup consisted of a Xeuss 2.0 HR SAXS/WAXS instrument from Xenocs SAS (Grenoble, France) with a Cu Kα source and a detector PILATUS3 R 1 M from DECTRIS AG (Baden, Switzerland) which was placed at a distance of about 550 mm from the samples. Precise calibration of this distance was ensured using a standard of Ag behenate before each measurement. Through azimuthal integration, the scattering curves were obtained from the two-dimensional scattering images using the software Foxtrot from Synchrotron SOLEIL (Saint-Aubin, France).

### 2.3. Thermogravimetric analysis (TGA)

The sample for TGA was prepared by drying 0.360 mL of a 20 mg mL^−1^ AuNP dispersion in a crucible made from aluminum oxide. To remove any solvent and moisture, the sample was dried in a drying oven for 16 h at 30 °C and 50 mbar before being placed in an analyzer of type STA 449 F3 Jupiter from NETZSCH-Gerätebau GmbH (Selb, Germany). The sample was heated from room temperature to 1200 °C at a rate of 10 °C min^−1^ in synthetic dry air while the mass was continuously recorded from 50 °C onward.

### 2.4. Nanoimprinting

#### 2.4.1. Substrate cleaning

PET foil of type Melinex 401 CW from DuPont Teijin Films UK Ltd. (Redcar, United Kingdom) with a thickness of 75 μm was employed as imprinting substrate. This type of foil has one pure PET side and one which has been treated to slip more easily. We imprinted onto the pure PET side. Prior to use, the PET foil was cleaned in an ultrasonic bath in a custom-made cleaning rack, applying the following sequence of solvents: 5 min acetone, 5 min ethanol, and 5 min Milli-Q ultrapure water. The cleaned substrate was dried for 30 min at 60 °C, just below its glass transition temperature of 70–80 °C. Acetone removed greasy residues, ethanol and ultrapure water removed acetone (thereby increasing polarity); the ultrapure water dried without any trace.

#### 2.4.2. Polydimethylsiloxane (PDMS) imprinting stamp fabrication

PDMS imprinting stamps were fabricated in a two-step moulding process. In the first step, a lithographically produced silicon master from AMO GmbH (Aachen, Germany), carrying the desired pattern of the final imprinting stamp (parallel line channels, patterned area *A* ≈ 7 cm × 7 cm, pitch *p* ≈ 19.5 μm, channel width *w*_c_ ≈ 1.6 μm and channel depth *d*_c_ ≈ 4.2 μm), was used as a mould for a first PDMS replicate. This replicate was then used in a second step as the master mould for the actual imprinting stamp.

Both masters had to be silanized prior to the moulding procedure. Silanization of the silicon master was carried out without pre-treatment, while silanization of the PDMS master was performed after its plasma activation for 1.5 min using an oxygen plasma at 0.3 mbar in a low pressure plasma reactor of type Pico from Diener electronic GmbH & Co. KG (Ebhausen, Germany). Each silanization was performed inside a conventional glass desiccator with a snap-on vial cap containing 30 μL of (tridecafluoro-1,1,2,2-tetrahydrooctyl)trichlorosilane from abcr GmbH (Karlsruhe, Germany). The snap-on vial cap was shielded from the respective master to achieve uniform silanization. Firstly, the desiccator was flushed with Ar (the silane is sensitive to humidity), evacuated to 3 mbar, and disconnected from the vacuum pump. After 30 min at room temperature, the desiccator was slowly ventilated with air.

The pre-polymer and the cross-linker of a PDMS kit, Sylgard 184 from Dow Inc. (Midland, USA), were mixed in a 10 : 1 (w/w) ratio prior to degassing in a Speedmixer DAC 600.2 VAC-P from Hauschild GmbH & Co. KG (Hamm, Germany) at 2350 rpm and 1 mbar for 3 min. The resulting mixture was poured onto the respective master which carried a Teflon ring (2 mm thick) as spacer, sealed at the edges with add-i-gum light N from DE Healthcare Products (Gillingham, UK), and a glass plate as top sealing to ensure uniform thickness. The PDMS was cured for 3 h at 80 °C before carefully peeling off the replica along the stamp channels.

The replica of the PDMS master was cut into two equal halves, each being an imprinting stamp with an area of 3.5 cm × 8 cm of which 3.5 cm × 7 cm were patterned and 3.5 cm × 0.5 cm at either end were not. The latter served as run-in and run-out areas during imprinting. Larger areas can be printed using the complete replicate.^8^

#### 2.4.3. Nanoimprinting

For nanoimprinting, a patterned PDMS stamp was attached to a custom-made cylindric steel roller (3 kg, 8 cm in diameter and height) with a double-sided tape from tesa SE (Norderstedt, Germany). The weight of the roller provides the pressure needed for imprinting. We mounted the steel roller onto a modified TQC Sheen automatic film applicator from Industrial Physics Inks & Coatings GmbH (Hilden, Germany) which served as carrier. Right before imprinting, 60 μL of ink were injected directly in-between the attached PDMS stamp and the PET substrate using a pipette (continuous processing is possible *e.g.* with a syringe pump for the ink). During imprinting, the linear movement of the carrier (4 mm s^−1^) in direction of the stamp's parallel line-like channels is translated into a rolling motion of the steel roller over the substrate. A detailed description of the imprinting process is given in Maurer *et al.*^8^ and Kister *et al.*^7^ Before its reuse, ink residues adhering to the PDMS stamp after imprinting were removed using Scotch Magic Tape from 3M Deutschland GmbH (Kleinostheim, Germany). There was no degradation of the stamps apparent from the geometry of the prints after using the same stamp 60 times. To avoid capillary condensation during imprinting, it has to be executed well above the dew point. Typically, we imprinted at 22 °C and 55% *rH* (dew point of 12.5 °C) and stored the electrodes under the same conditions in the dark.

### 2.5. Plasma sintering

Plasma sintering was performed within a low pressure 13.56 MHz RF plasma reactor of type Pico from Diener electronic GmbH & Co. KG (Ebhausen, Germany) right after nanoimprinting. A mixture of 5% H_2_ in 95% Ar (H_2_/Ar) served as process gas. Plasma sintering was carried out at room temperature for 20 min at ≈ 0.3 mbar with 100 W RF power.

### 2.6. Sheet resistance measurements

The electrodes' sheet resistances *R*_sh_ were determined in 2-point-probe configuration with a multi-channel multi-meter (DAQ6510 data acquisition logging multi-meter system) which was equipped with multiplexer cards (7702 40-channel differential multiplexer module with screw terminals) from Keithley Instruments GmbH (Germering, Germany). A fast drying Ag paste ACHESON Ag DAG 1415 from Plano GmbH (Wetzlar, Germany) and AGF 1 miniature crocodile clamps from SKS Kontakttechnik GmbH (Niederdorf, Germany) were used for contacting. The Ag paste was deposited as two parallel lines, each 1.5 cm long and spaced 1.5 cm apart, forming a square measurement field. The measured resistances corresponded directly to the desired sheet resistances. Measurements were automatically recorded every 10 min during storage under ambient conditions (*T* ≈ 22 °C, *rH* ≈ 55%) in the dark to avoid *e.g.* radiation heating.

### 2.7. UV-vis spectroscopy

The electrodes' optical transmittances were determined using a Cary 5000 UV-vis-NIR spectrophotometer from Agilent Technologies Deutschland GmbH (Waldbronn, Germany) having a tungsten halogen light source for the visible and a deuterium arc light source for the UV range. The respective electrode on PET was mounted behind a blackened metal mask with circular aperture (5 mm in diameter). Measurements were carried out in the range 400 nm to 800 nm in double-beam mode against air at scan rates of 600 nm min^−1^. A PET substrate with the imprinted electrode on top was placed such that the blank side was in contact with the mask to prevent any damage to the electrode on the other side. Baselines of the bare PET substrates were recorded, too, as references.

### 2.8. Cross-sectioning *via* focused ion beam (FIB)

Cross-sections of printed and of sintered lines were prepared with a FEI Versa 3D DualBeam from Thermo Fisher Scientific GmbH (Schwerte, Germany). The surfaces of the lines were protected by layers of Pt which were first deposited using the electron beam, and then with an ion beam.

### 2.9. Transmission electron microscopy (TEM)

All FIB cuts were characterized at an acceleration voltage of 200 kV in a JEM 2010 from JEOL GmbH (Freising, Germany).

### 2.10. Scanning electron microscopy (SEM)

For SEM imaging, either a FEI Quanta 400 ESEM or a FEI Versa 3D DualBeam from Thermo Fisher Scientific GmbH (Schwerte, Germany) with secondary electron detectors was used. Instead of PET, polished p-type silicon wafers from Siegert Wafer GmbH (Aachen, Germany) were used as substrate for better imaging.

## 3. Results and discussion

**Fig. 1:**
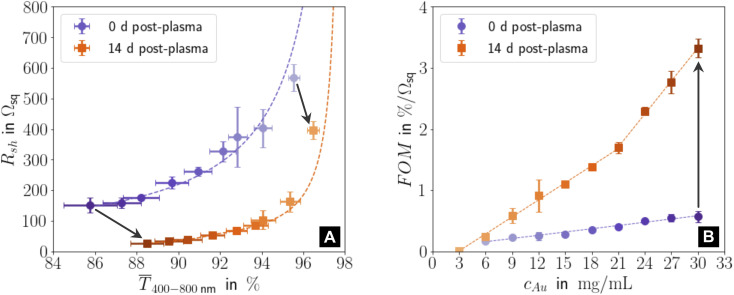
(A) Sheet resistances *R*_sh_ and mean optical transmittances *T̄*_400–800_ for electrodes directly after plasma sintering and fourteen days later. (B) Change of the figure of merit FOM = *T̄*_400–800_·*R*_sh_^−1^ with gold concentration *c*_Au_ for new and aged samples. All graphs show averages and standard deviations from three samples, and the corresponding fits (dashed lines). Fig. S1 in the ESI† has the complete data set and the fitting details for (A). Light colours represent lower *c*_Au_.

Fig. 1 illustrates the performance of freshly prepared (purple) and aged (orange) metal-grid FTEs printed using inks with different gold concentrations *c*_Au_ (denoted in the figure with the color gradient, where the intensity of the color indicates higher concentrations). We printed grids from oleylamine-capped gold nanospheres (AuNPs), plasma-sintered them, and compared their mean transmittances *T̄*_400–800_ in the visible range to their sheet resistances *R*_sh_ immediately after plasma sintering and two weeks later. Sheet resistances *R*_sh_ and optical transmittances *T̄*_400–800_ consistently decreased with increasing concentration *c*_Au_ in the range of 3–30 mg mL^−1^. Lower concentrations were insufficient to form percolating, conductive lines; higher concentrations led to optical transmittances of below 85%. The figure of merit according to Fraser and Cook,^3^

1 FOM = *T̄*_400–800_·*R*_sh_^−1^,

compares the ratio of *T̄*_400–800_ and *R*_sh_ at different concentrations *c*_Au_; it increased with *c*_Au_.

Resistances were more sensitive to *c*_Au_ than transmittances, and the FOM of aged electrodes was more sensitive to *c*_Au_ than that of freshly sintered ones. During 14 days of ageing, *T̄*_400–800_ (Fig. S3 and S4 in the ESI† show the averaged optical transmittance spectra for all concentrations immediately after plasma sintering and 14 days later) always increased (by up to ≈ 3.17%) while *R*_sh_ always dropped (by up to ≈ 82.2%), which increased the FOM up to ≈ 5.8 times. This is in stark contrast to the situation for metal grid FTEs printed with oleylamine-capped ultra-thin gold nanowires (AuNWs). Their sheet resistances increased by ≈30% in 14 days for concentrations up to 3 mg mL^−1^, and they became non-conductive within 8 h for *c*_Au_ ≥ 5 mg mL^−1^.^33^

We studied the ageing mechanisms to optimize the stability and performance of the affected FTEs. In the following, we give an overview of the resistances' temporal evolution, compare it to structural analyses, and develop mechanistic hypotheses.

**Fig. 2:**
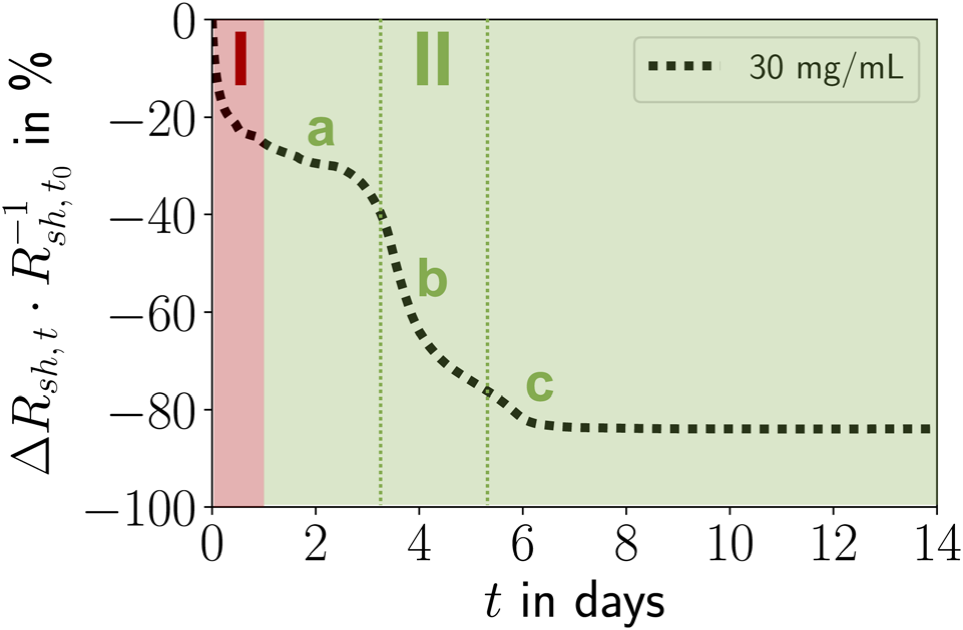
Stages I and II of electrode ageing: (I) post-plasma relaxation effects in and consolidation of the shell, (II) particle re-organization, de-mixing, coarsening, and densification of the core with Au plating onto the shell, followed by stabilization or (for *c*_Au_ < 15 mg mL^−1^) solid-state de-wetting. Stage II can be split into an initial phase a, an intermediate phase b, and a final phase c.

**Fig. 3:**
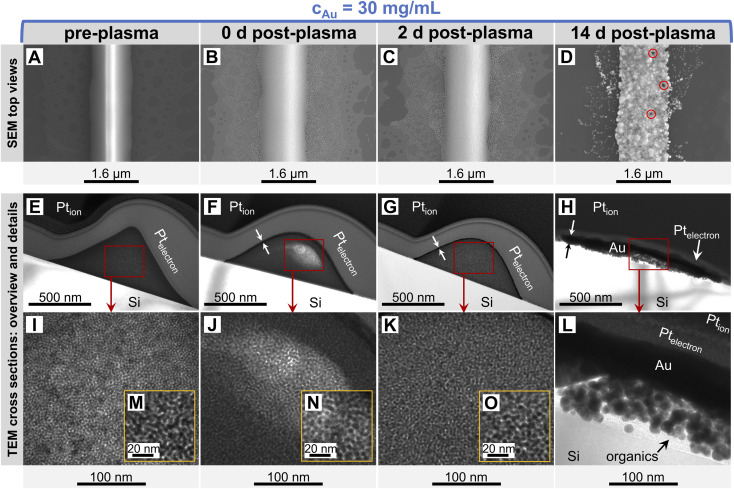
Surfaces and cross-sections of lines imprinted at 30 mg mL^−1^ before (A, E, I, M), immediately after (B, F, J, N), two days after (C, G, K, O), and fourteen days after (D, H, L) plasma sintering. They show the smooth line surface flanked by a thin layer of bleeded spheres (A) and the amorphous sphere arrangement in triangular shape (E, I and M) before, the now Gaussian-shaped core–shell structure (F, J and N) right after and (G, K and O) two days after as well as the densified core (H and L), the coarsened surface with occasional pores (red circles) and the coalesced bled spheres (D) fourteen days after plasma sintering. Note that each TEM image was taken from an individual TEM lamella. We prepared lamellae from different locations of the same FTE sample.

The relative change in sheet resistance (*R*_sh,*t*_ − *R*_sh,*t*_0__)·*R*_sh,*t*_0__^−1^ = Δ*R*_sh,*t*_·*R*_sh,*t*_0__^−1^ at time *t* progressed in two sequential stages. Their exact progressions depended on *c*_Au_, but the qualitative features were similar for all concentrations. Differences between the stages were particularly distinct for *c*_Au_ = 30 mg mL^−1^, the case shown in Fig. 2. An initial exponential decay (stage I) was followed by a reversed S-curve (stage II). We analyzed the morphological evolution of the lines and correlated it with the change in their resistance (Fig. 3).

The printed structure initially consisted of randomly and densely packed nanospheres. Small-angle X-ray scattering (SAXS, Fig. S6 of the ESI†) confirmed that the printed particles with a core diameter of *d*_sp_ = 3.7 ± 0.37 nm packed with a center-to-center distance of *a*_c–c_ = 5.61 ± 0.07 nm (Sections 3.1 and 3.2 in the ESI†). The surface-to-surface distance of the Au cores of *a*_s–s_ ≈ 1.91 nm was about the length of an oleylamine (OAm) molecule (*l*_OAm_ ≈ 2.05 nm (ref. 36)), probably due to interdigitation of the OAm shells.^37^

The printed lines were slightly narrower than the stamp feature *w*_c_ = 1.6 μm and had triangular cross-sections with a central ridge that appears as a bright center (Fig. 3A). Limited bleeding formed a sub-mono AuNP layer barely visible in the cross-section of Fig. 3E.

Plasma sintering blunted the ridge (Fig. 3B), reduced its height, and formed a conductive line with a Gaussian profile (Fig. 3F). Gold and OAm were moved or removed during plasma sintering. The outermost particles were sintered and formed a thin Au shell with a thickness of *t*_shell_ = 8.07 ± 1.36 nm.^23^ This shell was slightly thicker than that reported for plasma-sintered AuNW lines.^23^

Ageing stage I (Fig. 2) was not accompanied by visible morphological changes (Fig. 3M–O). The resistance in Fig. 2 indicates that ageing started immediately after plasma sintering, but there were no changes visible in electron microscopy (EM) that would explain the initial drop in *R*_sh_ during stage I. The unchanged microstructure and the time scale suggest that stage I is dominated by stress relaxation that reduces atomic lattice distortions in the metal shell.^38^ Slow consolidation processes likely close small pores in the shell, and metal grain sizes increase. The overall process can be likened to the final stage of ceramic solid-phase sintering.^39^ It is interesting to compare it to the ageing of AuNW grids at high *c*_Au_. These rapidly age and degrade after plasma sintering by fast de-mixing and compaction of the hybrid core, processes that damage the conductive shell and are clearly visible in EM.^33^ We find that plasma-sintered spherical nanoparticles form a thicker,^23^ less porous shell that shields the hybrid core from the plasma. This limits the removal of OAm and fragments thereof from the core that dominates the ageing of AuNW-derived structures.^33^

Ageing stage II was characterized by a reversed S-curve course that can be divided (Fig. 2) into an initial, an intermediate, and a final phase. The decrease of *R*_sh_ started slowly, accelerated, and finally stabilized. This may be compared to powder sintering with a liquid phase due to a low-melting secondary powder component, where most of the densification occurs quickly in the intermediate phase, too.^39^ A similar liquid phase may form in the hybrid core due to ”melting” of the ligand shells that are partially degraded by plasma sintering.

The decrease of resistance in stage II suggests coordinated particle movement inside the core rather than the motion of single particles that fill gaps. This is consistent with the average particle spacing from SAXS *a*_s–s_ that is ≈48% below the particle diameter *d*_sp_ and would not allow single particles to move inside packing. Instead, collective particle motion caused morphological changes that were clearly visible in electron microscopy Fig. 3D, H and L. The hybrid core had densified significantly, confirming the removal of a relevant fraction of OAm during plasma sintering. The surface of the lines had visibly coarsened after 14 days and pores were visible (indicated by red circles). We found that these pores appeared in lines printed at the same ink concentrations at similar densities, with spacings of at least 1 μm. This may indicate a “depletion” volume that forms below the pores. Metallic Au apparently migrated to the shell and increased its thickness from *t*_shell_ = 8.07 ± 1.36 nm (ref. 23) up to ≈ 100 nm (Fig. 3H and L). This migration probably decreased *R*_sh_ and stabilized the aged lines against solid-state dewetting.^40^ The shell thickness was surprisingly uniform across the entire width, indicating uniform plating from the core onto the inside of the shell. The aged lines appeared slightly wider than before, probably because the core partially collapsed in height with an intact shell. This reduced optical transmittance, an effect overcompensated by coalescence of the spheres in the thin layers surrounding the lines (Fig. 3D). This coalescence reduced optical scattering and probably explains the 3.17% increase in optical transmittance. Larger Au spheres formed in the core, probably by Ostwald ripening.^41^

**Fig. 4:**
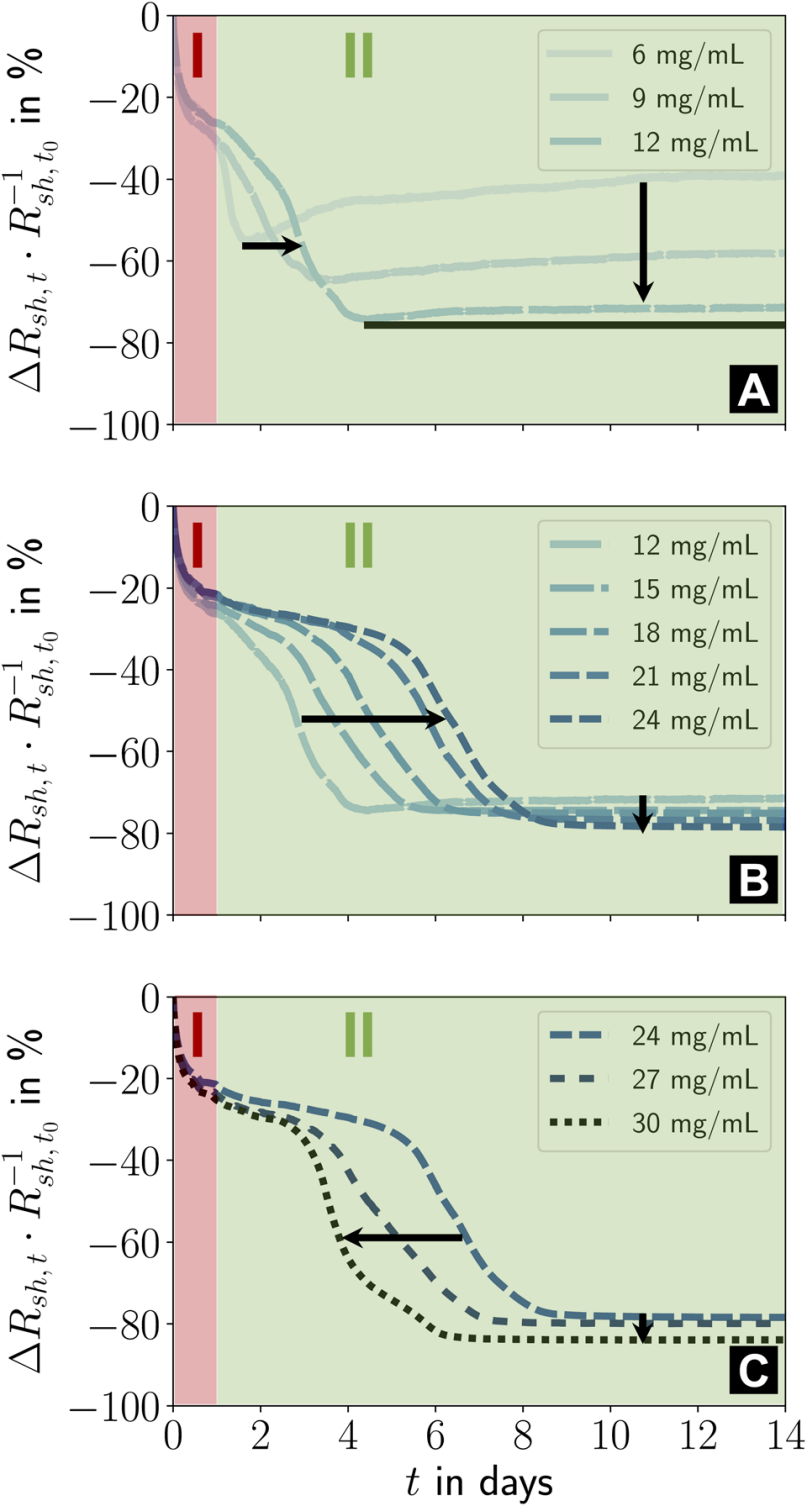
(A–C) Effect of ink concentration *c*_Au_ on the two ageing stages I and II. Relative changes in sheet resistance during fourteen days of storage under ambient conditions in the dark are shown. The different concentrations were distributed over three plots for improved readability. Figure S2 in the ESI† illustrates the repeatability and the result for *c*_Au_ = 3 mg mL^−1^.

The ageing of lines printed at lower *c*_Au_ was qualitatively similar (Fig. 4A–C). At *c*_Au_ = 6 mg mL^−1^ to 12 mg mL^−1^ (Fig. 4A), stage I was unchanged, but the intermediate phase of stage II started earlier than at *c*_Au_ = 30 mg mL^−1^ and caused a smaller relative decrease in *R*_sh_.

Previous studies reported that lines printed at low *c*_Au_ were up to ≈ 41% wider, lower in height, more porous, and had disconnected gold parts that did not contribute to conductance.^23^ Lines printed at higher *c*_Au_ had smoother surfaces and were higher and narrower (minimum width at *c*_Au_ = 12 mg mL^−1^, Fig. 4B). Our recent results suggest that porous, flat, wide lines age unfavorably. The conductance decrease in stage II at *c*_Au_ = 6 mg mL^−1^ strongly reminds of the ageing of wire-based electrodes^33^ imprinted at low concentrations that have a rough, porous surface and slowly degrade due to solid-state de-wetting.^40^

Lines printed at *c*_Au_ = 12 mg mL^−1^ to 24 mg mL^−1^ (Fig. 4B) aged in similar ways. Stage II lasted longer with increasing *c*_Au_. The (stable) resistance in its final phase consistently decreased with increasing *c*_Au_, while its relative change increased due to the growing Au fraction in the hybrid core. The initial steep drop in relative *R*_sh_ remained unchanged, consistent with a relaxation mechanism. It is conceivable that the thin and wide lines that form at *c*_Au_ ≤ 9 mg mL^−1^ (ref. 23) promote the re-arrangement of particles in the hybrid core, obscuring the initial phase of stage II. A higher fraction of fragmented ligands may reduce viscosity and increase mobility.

Lines printed at *c*_Au_ = 24 mg mL^−1^ to 30 mg mL^−1^ (Fig. 4C) had similar stages I and II, but the length of the initial phase of stage II decreased with *c*_Au_. It appears likely that the residual organics inside the hybrid core reach a critical concentration as the line height grows linearly with *c*_Au_, while the plasma penetration depth is limited. The low binding energy of Au–NH_2_,^42,43^ the partial fragmentation of OAm during plasma sintering, and the low viscosity^44^ of OAm at room temperature increase the mobility of the core components^45–47^ such that particle re-orientation accelerates. This is consistent with FTEs imprinted from AuNWs, where the fraction in residual organics inside the hybrid core controlled the ageing rate.^33^

The ageing mechanisms discussed above explain the evolution of the FOM with *c*_Au_ after 14 days shown in Fig. 1B. The increase in the gold fraction of the compact shell with *c*_Au_ increased the overall conductance. The change in the slope δFOM/δ*c*_Au_ at 21 mg mL^−1^ (“kink”) indicates that a larger fraction of the AuNPs within the core migrated to the conductive shell for *c*_Au_ > 21 mg mL^−1^ than for *c*_Au_ ≤ 21 mg mL^−1^, probably due to their larger residual organic content that facilitates de-mixing. The kink in Fig. 1B coincides with the decreasing duration of the initial phase of stage II at approximately 21 mg mL^−1^.

The effect of the residual organic concentration inside the hybrid core plausibly explains why ageing in stage II consistently accelerates, too. Any Au plated from the core onto the shell increases the fraction of residual organics in the core. Mobile gold in hybrid phases that enables low-temperature annealing has been previously reported, including the mobility-increasing effect of plasmas.^48–50^

## 4. Conclusions

Flexible transparent electrodes were imprinted from AuNPs at different particle concentrations and plasma sintered, partially removing the OAm ligands. We found strong correlations between the ink concentration, the electrodes' ageing, and the resulting opto-electronic performance.

Plasma sintering formed conductive lines with an outer Au shell that covered a hybrid core. Organic residues in the core caused it to re-organize, de-mix, coarsen, and compact. The shell thickened as metallic gold from the hybrid core deposited on it. Residual organics facilitated de-mixing and enabled further densification of the core.

The change in resistance during ageing was characterized by two sequential stages at all ink concentrations. Stage I comprised post-plasma relaxation in and consolidation of the shell. Stage II comprised the re-organization, de-mixing, coarsening, and densification of the core as well as Au plating from the core onto the shell. It ended with a stable resistance at *c*_Au_ ≥ 15 mg mL^−1^ or solid-state de-wetting that degraded lines printed at lower concentrations.

Ageing in stage I was largely concentration independent, while stage II was clearly dependent on *c*_Au_ but always followed an inverted “S” shape. The largest relative decrease in *R*_sh_ within its intermediate phase of 40% to 50% within 2 to 3 days occurred at *c*_Au_ > 12 mg mL^−1^. The evolution is consistent with a liquid-like organic phase in the hybrid core not unlike the liquid phase in powder sintering after the liquefaction of a low-melting secondary powder component.^39^ The organic phase promoted plating of metallic Au onto the shell. This thickened the shell up to 100 nm and caused the FOM in the aged state to increase rapidly: δFOM/δ*c*_Au_ increased, reaching the highest FOM ≈ 3.32%·Ω_sq_^−1^ at *c*_Au_ = 30 mg mL^−1^. Ink concentrations of 6 mg mL^−1^ to 24 mg mL^−1^ yielded thicker lines. This decreased the fraction of low-viscosity ligand fragments, increasing the duration of stage II.

The results confirm the importance of a robust shell during ageing. The concentration of residual organics and the ratio between particle spacing and core diameter set the kinetics of gold plating on the shell. Future studies could assess whether temperature can be used to control the ageing kinetics, and whether an optimal ratio of particle diameter to spacing exists.

## Author contributions

L. F. Engel designed the experiments, performed the experiments himself or supervised them, implemented Python code for data analysis and visualization, analysed and visualized all acquired data and wrote the original draft. T. Kraus and L. González-García devised the research strategy, acquired funding, administered, and supervised the project. They reviewed, edited, and commented the manuscript drafts.

## Conflicts of interest

There are no conflicts to declare.

## Supplementary Material

NA-005-D3NA00293D-s001

## References

[cit1] Kim G. H., Woo H., Kim S., An T., Lim G. (2020). RSC Adv..

[cit2] Xu G., Li Y. (2020). Nano Select.

[cit3] Fraser D. B., Cook H. D. (1972). J. Electrochem. Soc..

[cit4] Lu X., Zhang Y., Zheng Z. (2021). Adv. Electron. Mater..

[cit5] Maurer J. H. M., González-García L., Reiser B., Kanelidis I., Kraus T. (2016). Nano Lett..

[cit6] Woerle J., Rost H. (2011). MRS Bull..

[cit7] Kister T., Maurer J. H. M., González-García L., Kraus T. (2018). ACS Appl. Mater. Interfaces.

[cit8] Maurer J. H. M., González-García L., Backes I. K., Reiser B., Schlossberg S. M., Kraus T. (2017). Adv. Mater. Technol..

[cit9] Ko S. H., Park I., Pan H., Grigoropoulos C. P., Pisano A. P., Luscombe C. K., Fréchet J. M. J. (2007). Nano Lett..

[cit10] Park I., Ko S. H., Pan H., Grigoropoulos C. P., Pisano A. P., Fréchet J. M. J., Lee E.-S., Jeong J.-H. (2008). Adv. Mater..

[cit11] Park I. , Ko S. H., Pan H., Pisano A. P. and Grigoropoulos C. P., Proceedings of IMECE2007, 2007, 307–314

[cit12] Agrawal H., Garnett E. C. (2020). ACS Nano.

[cit13] Sciacca B., Berkhout A., Brenny B. J. M., Oener S. Z., van Huis M. A., Polman A., Garnett E. C. (2017). Adv. Mater..

[cit14] Reiser B., González-García L., Kanelidis I., Maurer J. H. M., Kraus T. (2016). Chem. Sci..

[cit15] Niittynen J., Abbel R., Mäntysalo M., Perelaer J., Schubert U. S., Lupo D. (2014). Thin Solid Films.

[cit16] Qi W. H., Wang M. P. (2004). Mater. Chem. Phys..

[cit17] Zhang Y., Zhang J., Lu Y., Duan Y., Yan S., Shen D. (2004). Macromolecules.

[cit18] Poulis J. A., Cool J. C., Logtenberg E. (1993). Int. J. Adhes. Adhes..

[cit19] Greene W. M., Oldham W. G., Hess D. W. (1988). Appl. Phys. Lett..

[cit20] Shaw S., Yuan B., Tian X., Miller K. J., Cote B. M., Colaux J. L., Migliori A., Panthani M. G., Cademartiri L. (2016). Adv. Mater..

[cit21] Reinhold I., Hendriks C. E., Eckardt R., Kranenburg J. M., Perelaer J., Baumann R. R., Schubert U. S. (2009). J. Mater. Chem..

[cit22] Ma S., Bromberg V., Liu L., Egitto F. D., Chiarot P. R., Singler T. J. (2014). Appl. Surf. Sci..

[cit23] Engel L. F., González-García L., Kraus T. (2022). Nanoscale Adv..

[cit24] Deignan G., Goldthorpe I. A. (2017). RSC Adv..

[cit25] Jiu J., Wang J., Sugahara T., Nagao S., Nogi M., Koga H., Suganuma K., Hara M., Nakazawa E., Uchida H. (2015). RSC Adv..

[cit26] Khaligh H. H., Goldthorpe I. A. (2013). Nanoscale Res. Lett..

[cit27] Madeira A., Plissonneau M., Servant L., Goldthorpe I. A., Tréguer-Delapierre M. (2019). Nanomaterials.

[cit28] Mayousse C., Celle C., Fraczkiewicz A., Simonato J.-P. (2015). Nanoscale.

[cit29] Song T.-B., Rim Y. S., Liu F., Bob B., Ye S., Hsieh Y.-T., Yang Y. (2015). ACS Appl. Mater. Interfaces.

[cit30] Rayleigh L. (1878). Proc. London Math. Soc..

[cit31] Maurer J. H. M., González-García L., Reiser B., Kanelidis I., Kraus T. (2015). ACS Appl. Mater. Interfaces.

[cit32] Maurer J. H. M., González-García L., Reiser B., Kanelidis I., Kraus T. (2016). Phys. Status Solidi A.

[cit33] Engel L. F., González-García L., Kraus T. (2022). Nanoscale Adv..

[cit34] Schubert U. , Hüsing N. and Laine R. M., Materials Syntheses: A Practical Guide, Springer, Wien, 2008

[cit35] Wu B.-H., Yang H.-Y., Huang H.-Q., Chen G.-X., Zheng N.-F. (2013). Chin. Chem. Lett..

[cit36] Mourdikoudis S., Liz-Marzán L. M. (2013). Chem. Mater..

[cit37] Reiser B., Gerstner D., González-García L., Maurer J. H. M., Kanelidis I., Kraus T. (2016). Phys. Chem. Chem. Phys..

[cit38] Rossnagel S. M., Cuomo J. J. (1989). Thin Solid Films.

[cit39] Salmang H. , Scholze H. and Telle R., Keramik, Springer, Dordrecht, 7th edn, 2007

[cit40] Thompson C. V. (2012). Annu. Rev. Mater. Res..

[cit41] Ostwald W. (1897). Z. Phys. Chem..

[cit42] Tang Q., Jiang D.-e. (2017). Chem. Mater..

[cit43] Shen H., Niu J., Li X., Wang H., Xing M., Chen X., Li L. S. (2012). Nanoscale.

[cit44] CRC Handbook of chemistry and physics: A ready-reference book of chemical and physical data, ed. W. M. Haynes, CRC Press, Boca Raton and London and New York, 97th edn, 2017

[cit45] Luedtke W. D., Landman U. (1996). J. Phys. Chem..

[cit46] Tian G., Zhao T., Niu J., Shen H., Li L. S. (2014). RSC Adv..

[cit47] Zhang Y., Yu P., Qi Y., Chen F., Li Y., Zhang Y. (2017). Mater. Lett..

[cit48] Ingham B., Lim T. H., Dotzler C. J., Henning A., Toney M. F., Tilley R. D. (2011). Chem. Mater..

[cit49] Volkman S. K., Yin S., Bakhishev T., Puntambekar K., Subramanian V., Toney M. F. (2011). Chem. Mater..

[cit50] Wünscher S., Stumpf S., Perelaer J., Schubert U. S. (2014). J. Mater. Chem. C.

