# Supplementary material for: Consolidation and performance gains in plasma-sintered printed nanoelectrodes
Source: Nanoscale Adv. 2023 Jul 11;5(16):4124–32. doi: 10.1039/d3na00293d (PMC10408613; doi:10.1039/d3na00293d)
Supplement: NA-005-D3NA00293D-s001 [file NA-005-D3NA00293D-s001.pdf]

Electronic Supplementary Material (ESI) for Nanoscale Advances.
This journal is © The Royal Society of Chemistry 2023

SUPPORTING INFORMATION TO: Consolidation and performance gains in plasma-sintered printed nanoelectrodes

Lukas F. Engel,[†] Lola González-García,[*,†,‡] and Tobias Kraus[*,†,¶]

†*INM — Leibniz Institute for New Materials, Campus D2 2, 66123 Saarbrücken, Germany*

‡*Department of Materials Science and Engineering, Saarland University, Campus D2 2, 66123 Saarbrücken, Germany*

¶*Colloid and Interface Chemistry, Saarland University, Campus D2 2, 66123 Saarbrücken, Germany*

E-mail: lola.gonzalez-garcia@leibniz-inm.de; tobias.kraus@leibniz-inm.de

1 Comparing optical transmittance and electric conductance of different grids

We determined the sheet resistance R_{sh} as resistance R of a quadratic electrode section with edge length $l = 15\,000\,\mu m$, used Ag paste for contacting and miniature crocodile clamps for two-point probe measurements. The square area contained 750 conducting lines. Its anisotropic sheet resistance was

$$R_{sh}^{-1} = R^{-1} = G = \sigma_{shell} \cdot \frac{750 \cdot A_{shell}}{l}, \qquad (S1)$$

measured parallel to the lines, with G being the conductance of the 750 lines in parallel.

To restrict the influence of R_{sh} data points at the lowest concentration c_{Au} (they were off-the-charts) when fitting the trade-off between sheet resistance and optical transmittance, the relation above was used for fitting $R_{sh}^{-1} = G$ vs. $\overline{T}_{400-800\,\mathrm{nm}}$ instead of R_{sh} vs. $\overline{T}_{400-800\,\mathrm{nm}}$. Thus, the fits in Fig. S1B correspond to the inverse of the linear fits in Fig. S1A.

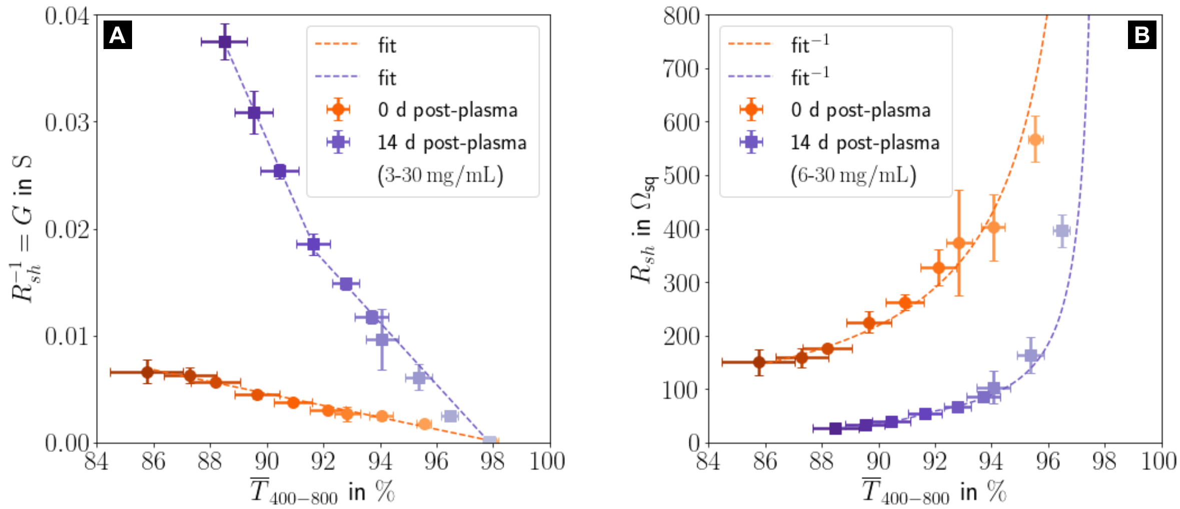

Figure S1: Trade-off between **(A)** conductance $G = R_{sh}^{-1}$ and optical transmittance $\overline{T}_{400-800}$ as well as **(B)** between sheet resistance R_{sh} and optical transmittance $\overline{T}_{400-800}$ for electrodes right after plasma sintering (initial state) and fourteen days later (aged state). The data points for $c_{Au} = 3\,\mathrm{mg/mL}$ have been omitted in B due to off-the-charts high R_{sh}. | The graphs show averaged values from three measurements each, the corresponding standard deviation as well as a fit (dashed lines). Light colours indicate lower c_{Au}.

2 Quantification of electrode ageing

We tracked the sheet resistance of AuNP electrodes imprinted at ink concentrations ranging from 3 mg/mL to 30 mg/mL on PET foil over time to investigate their ageing behaviour. Nanoimprinting at 3 mg/mL resulted in structures near the percolation threshold, exhibiting initial sheet resistances which were too high for FTEs.

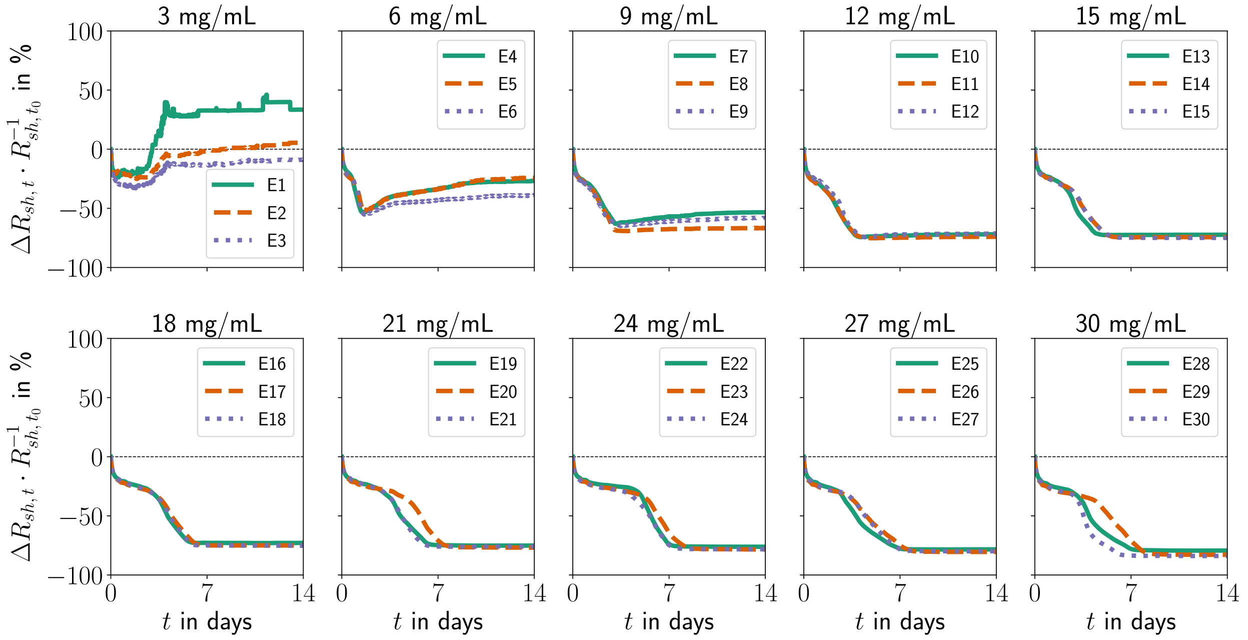

Figure S2: Relative change in sheet resistance $(R_{sh,t} - R_{sh,t_0}) \cdot R_{sh,t_0}^{-1} = \Delta R_{sh,t} \cdot R_{sh,t_0}^{-1}$ with time t for three electrodes each, nanoimprinted at the respectively same concentration c_{Au}.

In addition, we measured the optical transmittance spectra of the FTEs immediately after plasma sintering and 14 days later for all concentrations.

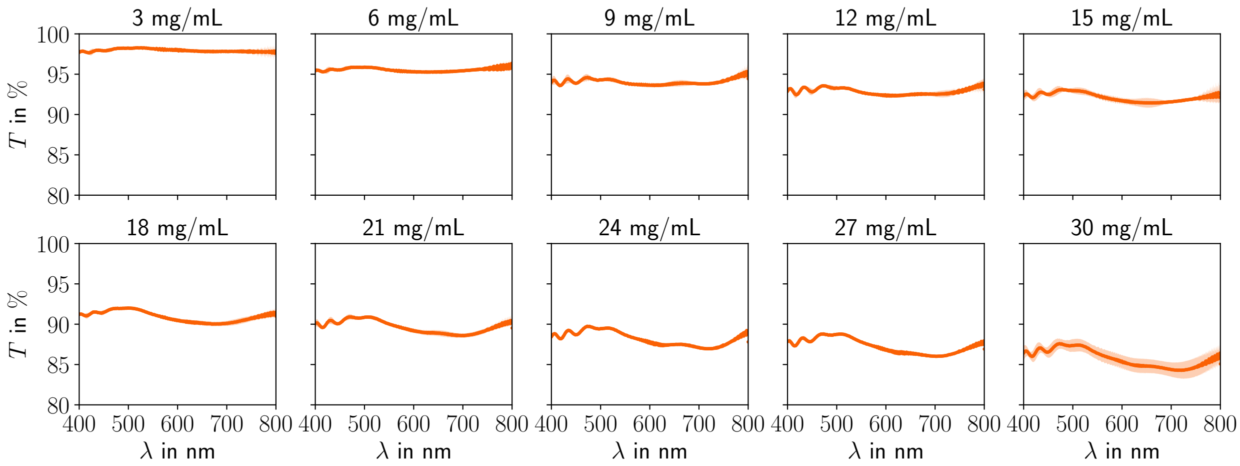

Figure S3: Optical transmittance spectra of the FTEs immediately after plasma sintering. Each graph shows the average from measurements on three FTEs imprinted at the same concentration c_{Au} as well as the standard deviation.

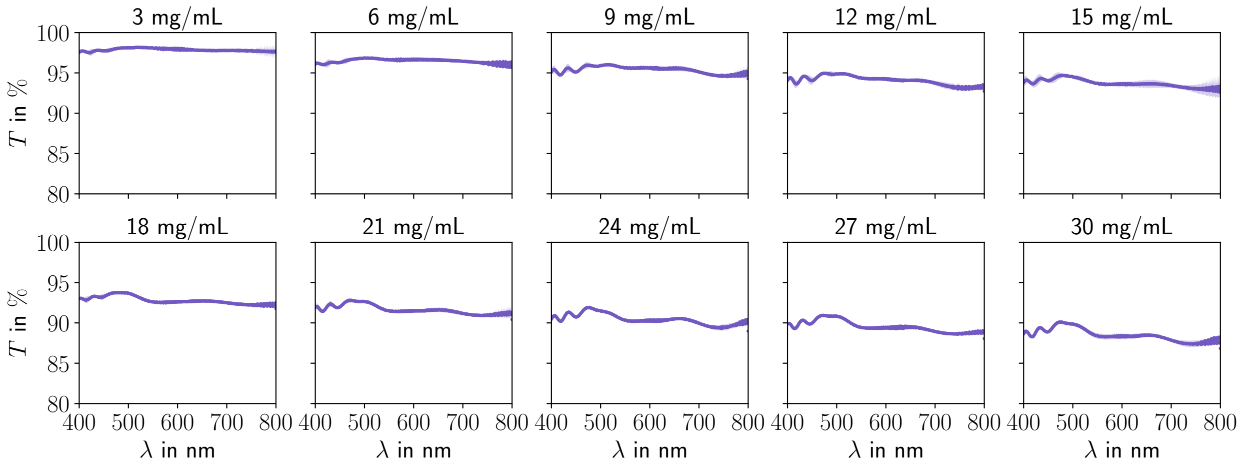

Figure S4: Optical transmittance spectra of the FTEs 14 days after plasma sintering. Each graph shows the average from measurements on three FTEs imprinted at the same concentration c_{Au} as well as the standard deviation.

3 Sphere properties

3.1 Core sizes and dispersion states

SAXS was employed to study the AuNP dispersion state within the whole concentration range used for nanoimprinting and to determine the average AuNP core diameter d_{sp}.

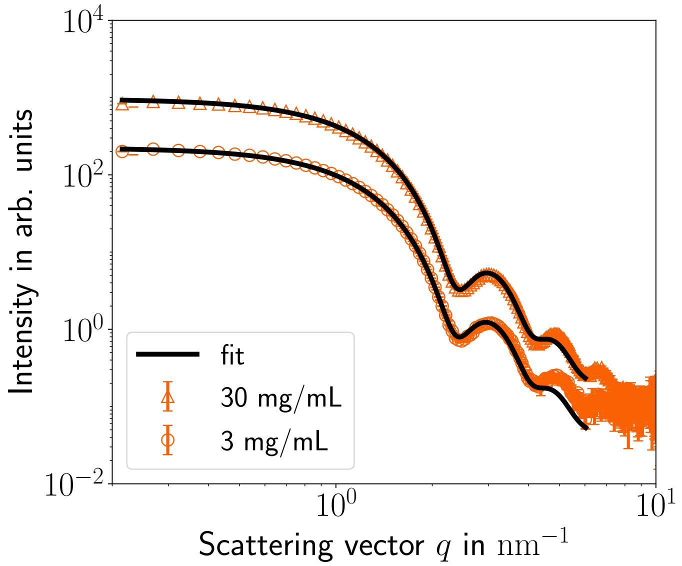

Figure S5: Radially integrated, fitted SAXS curves of the AuNPs used for nanoimprinting at both the highest (30 mg/mL) and the lowest concentration (3 mg/mL). The fits yield a core size of $d_{sp} = 3.7\,\mathrm{nm} \pm 10\,\%$. Agglomeration peaks are not present.

3.2 Core center-to-centre distance and sphere arrangement after imprinting

Small-Angle X-ray Scattering (SAXS) was employed to study the spheres' structural arrangement within lines nanoimprinted at $c_{Au} = 3\,\mathrm{mg/mL}$ and $30\,\mathrm{mg/mL}$. The single peak position q^* suggests an amorphous sphere arrangement. Based on the Ehrenfest relation,[1,2] the core center-to-center distance for spheres with a short range order (amorphous arrangement) corresponds to:

$$a_{c-c}^{amorph} = \frac{2\pi}{q^*} \cdot 1.23 = 5.61 \pm 0.07\,\mathrm{nm} \tag{S2}$$

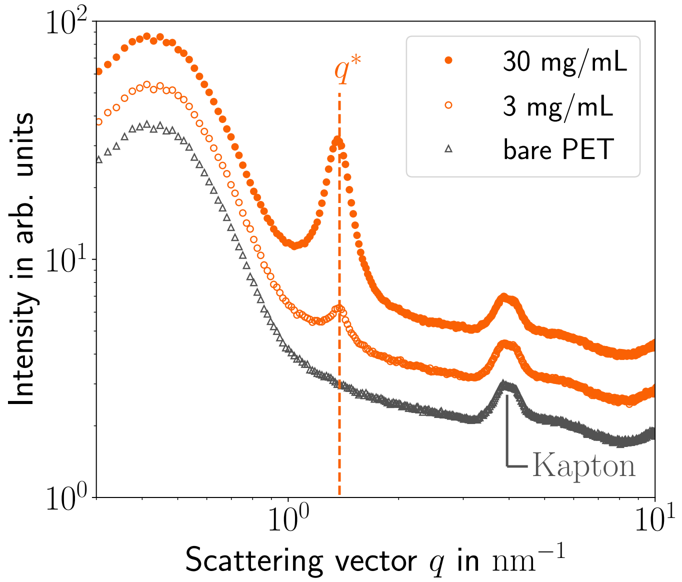

Figure S6: Radially integrated SAXS curves of electrodes nanoimprinted at 30 mg/mL (highest concentration) and 3 mg/mL (lowest concentration) on PET as well as of the bare PET as reference (the Kapton® peak is due to the Kapton® window separating sample and evacuated scattering path | scattering curves are shifted for improved visibility).

3.3 Organic content

The organic content of the synthesized and twice-purified Au nanospheres was determined with Thermogravimetric Analysis (TGA) and amounted to 16.98 wt% (see Fig. S7). This corresponds to a volume fraction of:

$$f_{V,OAm} = \frac{f_{w,OAm} \cdot (\rho_{OAm})^{-1}}{f_{w,OAm} \cdot (\rho_{OAm})^{-1} + f_{w,Au} \cdot (\rho_{Au})^{-1}} \cdot 100\,\% \approx 80.79\,\mathrm{vol\%} \tag{S3}$$

with $f_{V,OAm}$ the volume fraction of OAm, $f_{w,OAm} \approx 15\,\mathrm{wt\%}$ the mass fraction of OAm, $f_{w,Au} \approx 85\,\mathrm{wt\%}$ the mass fraction of Au, $\rho_{OAm} = 0.81\,\mathrm{g/cm^3}$ the density of OAm[3] and $\rho_{Au} = 19.3\,\mathrm{g/cm^3}$ the density of Au,[4] both at room temperature.

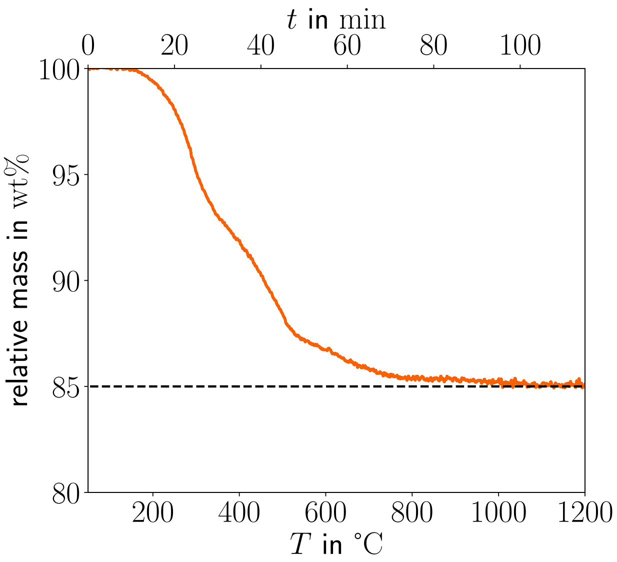

Figure S7: TGA of the synthesized and twice purified AuNPs.

References

1. Guinier, A.; Fournet, G. *Small-Angle Scattering of X-Rays*; John Wiley & Sons, Inc.: New York, 1955.

2. Feng, R.; Stachurski, Z. H.; Rodriguez, M. D.; Kluth, P.; Araujo, L. L.; Bulla, D.; Ridgway, M. C. X-ray scattering from amorphous solids. *Journal of Non-Crystalline Solids* **2014**, *383*, 21–27, DOI: `10.1016/j.jnoncrysol.2013.04.070`.

3. Oleylamine, approximate C18-content 80-90%: SDS. 2009; `https://www.fishersci.de/store/msds?partNumber=10400380&productDescription=100ML+Oleylamine%2C+approximate+C18-content+80-90%25&countryCode=DE&language=de`.

4. Haynes, W. M., Ed. *CRC Handbook of chemistry and physics: A ready-reference book of chemical and physical data*, 97th ed.; CRC Press: Boca Raton and London and New York, 2017.
